# Annotated dataset for training deep learning models to detect astrocytes in human brain tissue

**DOI:** 10.1038/s41597-024-02908-x

**Published:** 2024-01-19

**Authors:** Alex Olar, Teadora Tyler, Paulina Hoppa, Erzsébet Frank, István Csabai, Istvan Adorjan, Péter Pollner

**Affiliations:** 1 Eötvös Loránd University, Department of Physics of Complex Systems, Budapest, Hungary; 2 Eötvös Loránd University, Doctoral School of Informatics, Budapest, Hungary; 3 Semmelweis University, Department of Anatomy, Histology and Embryology, Budapest, Hungary; 4 Semmelweis University, Data-Driven Health Division of National Laboratory for Health Security, Health Services Management Training Centre, Budapest, Hungary

**Keywords:** Data publication and archiving, Machine learning

## Abstract

Astrocytes, a type of glial cell, significantly influence neuronal function, with variations in morphology and density linked to neurological disorders. Traditional methods for their accurate detection and density measurement are laborious and unsuited for large-scale operations. We introduce a dataset from human brain tissues stained with aldehyde dehydrogenase 1 family member L1 (ALDH1L1) and glial fibrillary acidic protein (GFAP). The digital whole slide images of these tissues were partitioned into 8730 patches of 500 × 500 pixels, comprising 2323 ALDH1L1 and 4714 GFAP patches at a pixel size of 0.5019/pixel, furthermore 1382 ADHD1L1 and 311 GFAP patches at 0.3557/pixel. Sourced from 16 slides and 8 patients our dataset promotes the development of tools for glial cell detection and quantification, offering insights into their density distribution in various brain areas, thereby broadening neuropathological study horizons. These samples hold value for automating detection methods, including deep learning. Derived from human samples, our dataset provides a platform for exploring astrocyte functionality, potentially guiding new diagnostic and treatment strategies for neurological disorders.

## Background & Summary

Astrocytes are types of glial cells which play essential roles in synapse modulation, homeostasis maintenance, and energy metabolism in the central nervous system (CNS)^1^. These star-shaped cells not only maintain the proper environment for neuronal signaling but also play a vital role in forming and regulating the blood-brain barrier^1^. Astrocytes undergo physiological and morphological changes in response to brain injury or pathological processes. Therefore, they have been implicated in a wide range of neurological disorders, such as Alzheimer’s disease, Parkinson’s disease, and Multiple Sclerosis^2^.

Accurate detection, segmentation, and quantification of astrocytes in brain tissue is fundamental when investigating the cellular mechanisms underlying astrocyte functions and their involvement in CNS disorders. As their morphology and local density serve as a histopathological biomarker, their quantification is an extremely important and common task in the field of pathology and neuroscience. However, traditional methods of astrocyte detection (involving manual cell counting) are time-consuming and subjective. This limits the ability to perform large-scale and reproducible quantitative analyses. The complex and versatile morphology of astrocytes makes automatic cell detection - as with the widely used ImageJ^3^ program - challenging and unreliable.

Recently, deep learning has emerged as a powerful approach for automated detection and segmentation of astrocytes in histology images. Deep learning algorithms are usually trained on large datasets^4–6^ of annotated signals (images, text, sound, etc.), enabling deep neural networks to learn complex features and patterns that are difficult for traditional methods to detect.

A few publicly available datasets have already been established to facilitate the development and evaluation of deep learning-based astrocyte detection algorithms^7,8^. These datasets provide high-quality images of astrocytes in the rat and mouse brain, along with manual annotations indicating the location and extent of each astrocyte in the image. For example, the BBBC042v1^7,9,10^ dataset includes high-resolution images of astrocytes in the rat brain. These have been manually annotated by experts, ensuring high accuracy and reliability of the annotations. However, a major limitation of these datasets is their lack of scale and diversity of samples^9,11^, which would be necessary for exploring individual variability. This limitation is a general characteristic of datasets in this field. Our dataset stands out, offering samples from 8 different human patients, 16 tissue slides, and two different histochemical stains with widely used astroglia markers: glial fibrillary acidic protein (GFAP) and aldehyde dehydrogenase 1 family member L1 (ALDH1L1) Table 1. Furthermore, our annotations are on a scale larger than any previous dataset.

**Table 1:** Demographic characteristics of donors, including unique IDs for each case (Patient ID), age (years), sex (female/male), post-mortem interval (PMI; hours), source (location and scanner) and cause of death.

#	WSI ID	Patient ID	Age	Sex	PMI (h)	Source	Cause of death
1)	ID26698 (ALDH1L1)	S10196	60	female	7.5	NBB, Leica Scanner	Septicaemia
2)	ID26707 (ALDH1L1); ID26774(GFAP)	S12002	55	male	7.25	NBB, Leica Scanner	Intestinal ischemia by thrombosis of the a. mesenterica superior
3)	ID26710 (ALDH1L1); ID26769 (GFAP)	S12071	57	female	7.17	NBB, Leica Scanner	Euthanasia (metastatic urothelial cancer)
4)	ID44082 (GFAP)	S11096	70	female	6.25	NBB, Leica Scanner	Pulmonary insufficiency
5)	ID44084 (GFAP)	S12059	78	female	4.58	NBB, Leica Scanner	Bronchopneumonia and metastatic neuroendocrine pancreas carcinoma
6)	ID26705 (ALDH1L1); ID26780 (GFAP)	S11081	55	male	7.5	NBB, Leica Scanner	Euthanasia (esophageal cancer)
7)	21_04_20_104_12_a_ALDH1L1_1; 21_05_11_104_12_I_GFAP_1; 21_04_20_NP104_12_a_GFAP_1; 21_05_11_104_12_I_ALDH1L_1	104_12	65	female	24	OBB, 3DHistech Scanner	Breast cancer
8)	21_03_21_100_2017_12_ALDH1L1_1; 21_05_11_100_2017_12_GFAP_1; 21_05_11_100_2017_29_ALDH1L1_1	100_2017	55	female	18.5	OBB, 3DHistech Scanner	NA

In some cases, at least 2 images were processed per donor (tissue stained for glial fibrillary acidic protein (GFAP) or ALDH1L1. The WSI ID column contains processed image IDs. NBB refers to the Netherlands Brain Bank while, OBB to the Oxford Brain Bank.

In recent years, several studies have demonstrated the effectiveness of deep learning-based methods for astrocyte detection^7^ and segmentation^8^, highlighting these methods’ potential to enhance understanding of astrocyte morphology, physiology and their role in CNS disorders. Although these datasets are mostly based on rodent tissue, they have already become important resources for the neuroscience community, because they enable researchers to scale up astrocyte identification in brain tissue, thereby promoting the development of new diagnostic and therapeutic approaches for pathological conditions. However, it is not trivial for algorithms trained on rodent data to work just as effectively on images from human tissue^9,12^. Here we introduce, to our knowledge, the largest annotated dataset for the detection and evaluation of astroglia in post mortem human brain tissue.

## Methods

### Staining and scanning

Formalin-fixed, paraffin-embedded tissue blocks were provided by the Netherlands Brain Bank (NBB; Netherlands Institute for Neuroscience, Amsterdam) and the Oxford Brain Bank (OBB; University of Oxford, John Radcliffe Hosptal, Oxford). The experiments were approved by the Research Ethics Committee of the Hungarian Medical Research Council (reference number 38711-1/2019/EKU). All material was collected from donors from whom written informed consent had been obtained for brain autopsy and use of material and clinical information for research purposes. 6 thick serial sections were cut from the blocks on a Leica microtome. Sections were immunohistochemically stained against GFAP and ALDH1L1 following the protocol described in detail elsewhere^13,14^. Briefly, sections were deparaffinized in xylene (2 × 5 minutes) and rehydrated in a decreasing graded alcohol series (absolute ethanol, 96%, 70% ethanol solutions), then treated with *H*_2_*O*_2_ solution (3% in phosphate buffered saline, pH 7.4) for 20 minutes to block endogenous peroxidase activity. Antigen retrieval was performed by placing the sections in the autoclave for 10 minutes on 121 °*C* in citrate buffer (pH 6). Then, slides were placed into Sequenza System coverplates and racks (Thermo Scientific, 72110017, 73310017) and incubated with primary antibodies for 1 h at room temperature. The following primary antibodies were used: rabbit anti-GFAP (diluted 1:500 in Tris-buffered saline with 0.1% Tween 20 detergent (TBST); DAKO, Z0334) and mouse anti-ALDH1L1 (diluted 1:1000 in TBST; EnCor Biotechnology Inc., MCA-4A12). After generously washing with TBST, sections were incubated with horseradish peroxidase-linked secondary antibody (Envision Kit, Dako, K-5007) for 1 hour and immune reactions were visualized by 3,3′-diaminobenzidine (DAB) (diluted 1:50 in DAB substrate, Envision Kit, Dako, K-5007) applied for 90 seconds. In this step, enzyme-catalyzed oxidation produces brown end-product at the target antigen, thus allowing visualization. Finally, hematoxylin nuclear counterstain was applied for 90 seconds, after which sections were left in tap water for 5 minutes. Sections were dehydrated in an increasing graded alcohol series followed by xylene and coverslipped with DePeX. No immune reaction was observed on negative control sections, on which no primary antibody was applied during the staining procedure.

Sections were digitized with a whole-slide scanner (20X magnification with Aperio ScanScope AT Turbo, Leica Biosystems, pixel size: 0.5019; 40X magnification with Pannoramic Flash Desk DX, 3DHistech, pixel size: 0.3557 at level 2).

### Generating patches

**Fig. 1:**
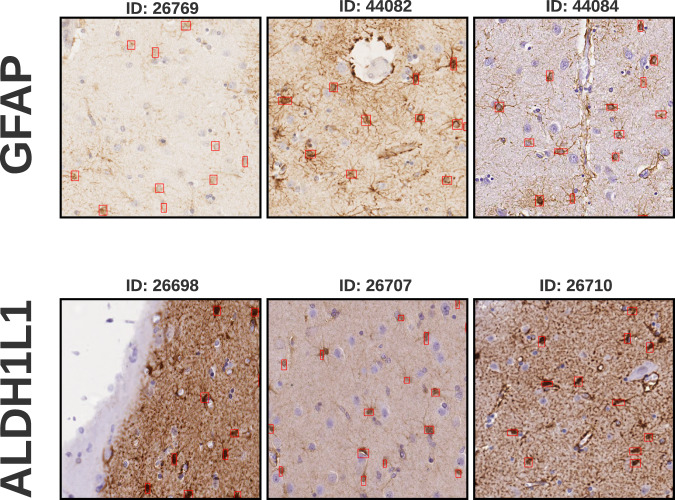
Multiple patches from different tissue samples that are present in our dataset and stained with different histopathological markers. The patches are arranged in a grid pattern, with each row corresponding to a different staining method. The top row shows patches stained with GFAP, presenting the large differences in staining between different slides. The second row shows patches stained with ALDH1L1. We can observe different cell surroundings and sizes between these patches from different whole-slides.

The image patches utilized in this study were created using the OpenSlide^15^ Python software. The alpha-channel, automatically added by the imaging software, was removed as the sole pre-processing step. No additional normalization or post-processing was performed. Subsequently, a subset of the patches was selected for annotation based on a pre-defined region of interest. The annotation process involved identifying and labeling glial cells with bounding-boxes (see Fig. 1). Annotators were required to enclose the glial cell body within the bounding box, but they weren’t obligated to include the entire cell along with its protrusions. Other specifications were not given as we wanted to capture the variability between annotators. The patches are suitable for downstream analyses, such as machine learning algorithms and data visualization techniques. These can provide insights into glial cell morphology, density, and inter-annotator variability.

### Annotation

**Fig. 2:**
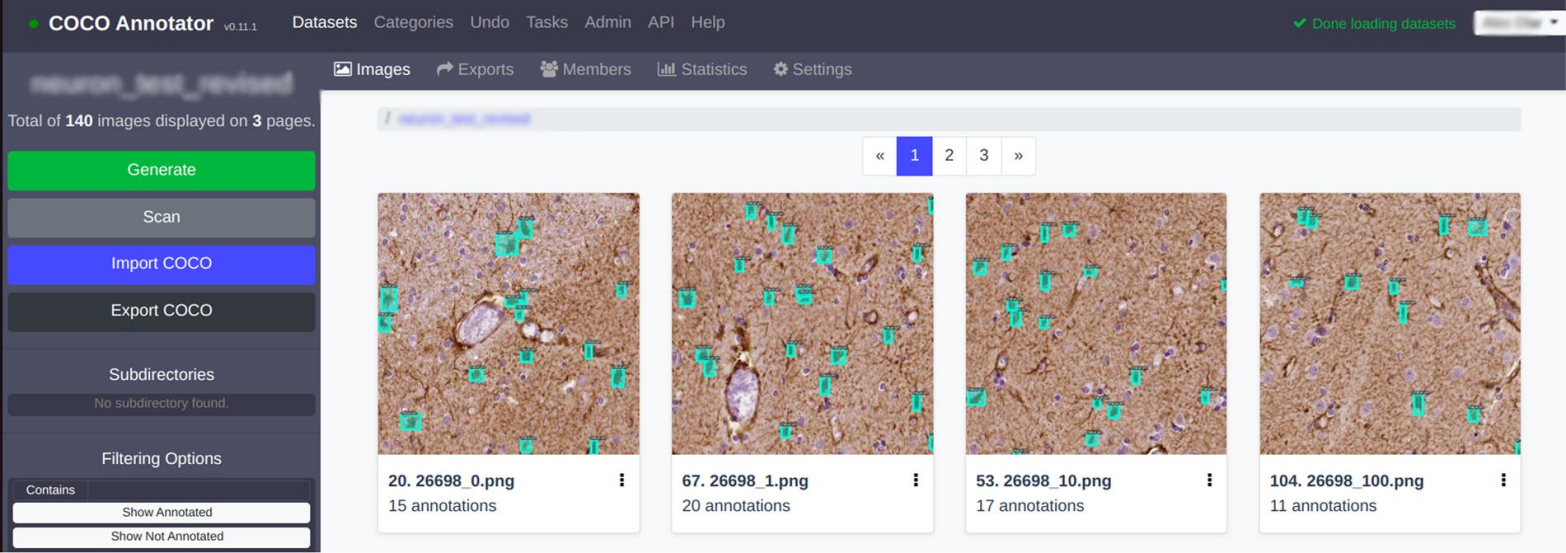
As shown in the illustration, the annotation process involves the application of various patches, each with its corresponding annotations. The coco-annotator tool simplifies this process by allowing users to create and manage annotations for each patch or region of interest, reducing the potential for errors or inconsistencies. In the accompanying statistics in Tables 1, 2, users can view details on the number of patches annotated, patient meta-data and other relevant metrics. By leveraging tools like coco-annotator, professionals can ensure the accuracy and consistency of their annotated data, leading to more reliable machine learning models and better overall results.

The annotation procedure was executed with a remotely available, modified local instance of the *coco-annotator* (https://github.com/jsbroks/coco-annotator/) tool. The process of annotating data is a critical step in machine learning, computer vision, and other related fields. It involves labeling and classifying various patches or regions of interest within an image or dataset, allowing for the creation of training data for machine learning algorithms. To ensure the accuracy and consistency of annotations, experts often use specialized tools like the open-source *coco-annotator*. The coco-annotator tool offers a user-friendly interface and a range of features that simplify the annotation process. With this tool, users can add annotations to images, including bounding boxes, segmentation masks, and key-points, with the option to save and load annotations as needed. Additionally, the tool includes a thumbnail viewer for easy reference and analysis (see Fig. 2). We deployed our local instance to the web and gave limited access to multiple annotators at different levels of expertise (see Table 2) to different subsets of the data. For our datasets, we have set up train and test splits with multiple annotations for the test sets in each staining category. We have created a ground truth annotation by the consensus of multiple annotators for the various test sets while each of them also annotated some of the sets individually - for details see Table 2. This resulted in a unique annotation set which mimics the real world scenario of different annotators creating slightly different annotations from each other. The training sets were annotated by experts but were not created with a consensus. However, we assume that any annotation noise averages out due to the large sizes of these sets. Our dataset also allows inter-observer variability study between annotators of the test sets which is unique regarding astrocyte machine learning datasets. All of the annotations are saved in the COCO format^5^. Along with the patched images, we’ve shared their original locations. These are referenced to the top-left corner position on the large whole-slide image (WSI), with both the width and the height of the patch. This metadata is contained within the file names. The original WSIs are also available with our dataset. The annotations are rectangular boxes around the astrocyte bodies; therefore, they can be used to train detection or weakly-supervised segmentation algorithms. Other than including the astrocyte body inside the bounding box, no specific requirement was given to annotators about labeling glial cells. However, annotators were informed not to necessarily include the lengthy protrusions of glial cells, as they could result in unreasonably large bounding boxes. In case of tightly clustered cells, the cell bodies were also annotated individually.

**Table 2:** Summary of glia cell annotations for two staining types, ALDH1L1 and GFAP, in our dataset consisting of train and test splits.

Staining	Split	# Images	Annotations/image	# Annotations	WSI ID	#Junior	#Mid-level	#Expert	Consensus	Pixel sizes
GFAP	train	4593	11.426	52480	21_05_11_100_2017_12_GFAP_1, 44082,26769	*	*	1	✗	0.5019, 0.3557, 0.3557
GFAP	test_5019_cohort_2	95	9.368	890	26780	1	2	*	✓	0.5019
GFAP	test_05019_cohort_1	200	4.294	803	44084, 26774	*	2	1	✓	0.5019, 0.5019
GFAP	test_03557	137	7.102	973	21_04_20_104_12_a_GFAP_1, 21_05_11_104_12_I_GFAP_1	1	2	*	✓	0.3557, 0.3557
ALDH1L1	train	3281	13.421	42989	21_05_11_100_2017_29_ALDH1L1_1, 26710,26707, 21_03_21_100_2017_12_ALDH1L1_1	*	*	1	✗	0.5019, 0.3557, 0.3557, 0.5019
ALDH1L1	test_05019_cohort_2	95	14.021	1332	26705	1	2	*	✓	0.5019
ALDH1L1	test_05019_cohort_1	140	13.957	1954	26698	3	2	1	✓	0.5019
ALDH1L1	test_03557	189	11.974	2263	21_05_11_104_12_I_ALDH1L_1, 21_04_20_104_12_a_ALDH1L1_1	1	3	*	✓	0.3557, 0.3557
	All	**8730**	**12.003**	**103684**	**16**					

The table shows the number of images and annotations per image, the total number of annotations, the unique IDs of the corresponding raw WSIs (whole-slide images), and the additional annotation files labeled by different junior (1 year experience), mid-level (2-5 years experience), and expert (5+ years experience) annotators. The last row contains the total number of images 8730, the average annotation count/image *12.003* and the very large amount of *103'684* total bounding boxes labeled in this work across 16 whole-slides. The pixel sizes are given in *μ*m/pixel units.

### Baseline models

In this section, we describe some detection baseline models we used for glia detection with the *mmdetection* framework^16^. We evaluated their performance using both the COCO metrics (https://cocodataset.org/#detection-eval) and the Free-Response Receiver Operating Characteristic (FROC) analysis. We conducted this analysis to showcase the dataset’s effectiveness with its diverse samples.

Our baseline is a Faster R-CNN^17^ model with the ResNet-50^18^ backbone functioning as feature-pyramid^19^ network. This model was chosen due to its popularity in object detection tasks and its availability in the *mmdetection* framework. We separately trained models on the ALDH1L1 and GFAP training sets and evaluated each on its respective test sets. These models serve merely as baselines, demonstrating the feasibility of using this dataset to generalize to new patients, different slides and various regions in a relatively heterogeneous environment.

The training procedure for the Faster R-CNN models involved several steps. First, the models were initialized with pre-trained weights on the COCO dataset. Next, we fine-tuned the models on our glia training datasets. Both stainings were handled separately using the stochastic gradient descent (SGD) optimizer. During training, data augmentation techniques such as random horizontal flipping and random resizing were applied to increase the variability of the data. The models were trained for 12 epochs, and the learning rate was reduced by a factor of 10 after 7 and 10 epochs, which is a standard procedure training detection models. Additionally, we used a batch size of 8 and initiated a learning-rate warm-up for the first 250 training steps. Finally, we selected the last model checkpoint and evaluated it on all the test sets using both the COCO metrics for a bounding box overlap and FROC analysis for a box-center position evaluation.

**Fig. 3:**
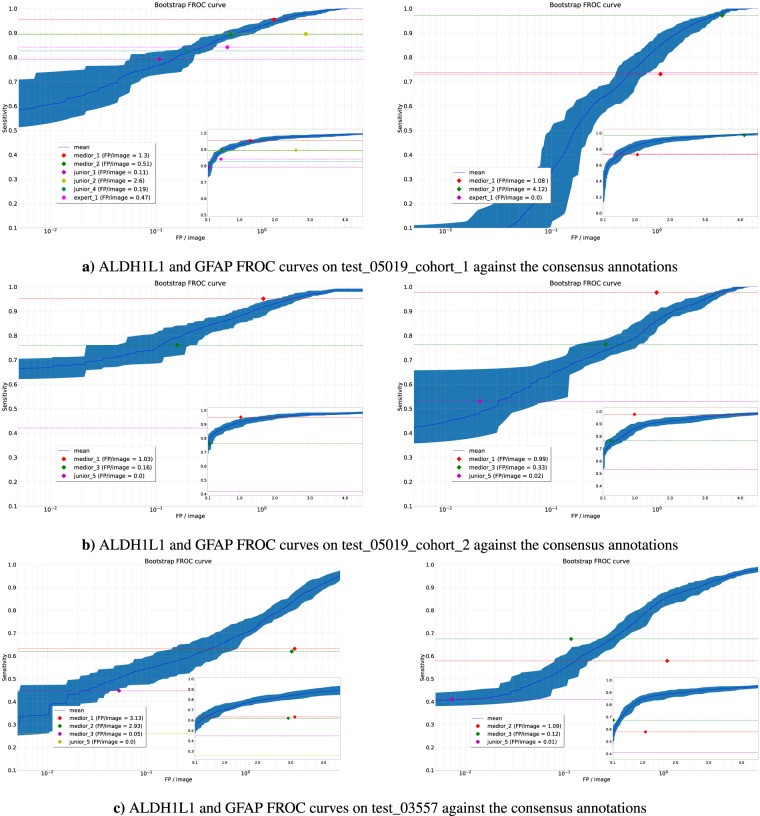
Bootstrapped FROC curve from the FasterRCNN model with the ResNet 50 backbone ran with the mmdetection framework trained on the ALDH1L1 (left) and GFAP (right) staining patches and evaluated on the test sets: test 05019 cohort 1, test 05019 cohort 2 and test 03557 respectively. The colored points indicate the human annotators also against the consensus who individually annotated the test sets and therefore could directly be compared with the machine’s output. We can observe that in most cases the tuned models are beating the human annotators compared to the consensus annotation. However, there are cases when experts and highly-specialized mid-level annotators beat the baseline.

For the COCO evaluation, we used the default mmdetection implementation of the COCO evaluation metric, which computes the Average Precision (AP) and the mean AP (mAP) for different intersection over union (IoU) thresholds^20^. Due to the significant variability in bounding box sizes, we adjusted the thresholds to range from 0.05 to 0.50. Our primary interest lies in positive detection rather than achieving perfect overlap. Intersection over union (IoU) scores are primarily relevant when seeking maximum overlap with similarly-sized detections. This does not apply to our situation since different annotators provided varying bounding box sizes for glial cells. For these reasons, we are transitioning from the IoU score to the well-established^21,22^ center-based approach in the subsequent sections. Nonetheless, we deemed it necessary to present the widely recognized COCO metrics, as an alternative evaluation approach. The FROC analysis computes the sensitivity at different levels of false positives per image (FPPI) and plots the curve of sensitivity versus FPPI^23^. A detection was considered correct if the center of the proposed prediction box fell within a consensus box as in previous works^21,22^. Our baseline models perform comparably to human annotators on all levels - at a specific FPPI rate for the ALDH1L1 and the GFAP staining - see Fig. 3 and Table 3. We share the COCO-metrics evaluated with the *cocoapi* library^5^ in Table 3. We also share our trained model weights and the detection results from the baseline models in addition to our dataset that can be plugged into the *mmdetection* framework easily.

**Table 3:** COCO-metric evaluated with the Python implementation of the *cocoapi*, the IoU (intersection over union) ranges have been modified as the IoU score is not extremely representative in our case since the sizes of the actual annotations do not matter much, just the ability to detect - that is why we are using the center-based ground truth matching in the FROC pipeline.

a) Average Precision (AP) and Average Recall (AR) scores for Faster-RCNN R50 ALDH1L1 test 05019 cohort 1.									
Metric			IoU			Area			Value
AP			0.05:0.50			all			**0.737**
AP			0.05:0.50			small			0.739
AP			0.05:0.50			medium			0.781
AR			0.05:0.50			all			**0.853**
AR			0.05:0.50			small			0.854
AR			0.05:0.50			medium			0.814
**b) Average Precision (AP) and Average Recall (AR) scores for Faster-RCNN R50 GFAP on test 05019 cohort 1**.									
**Metric**		**IoU**			**Area**			**Value**	
AP		0.05:0.50			all			**0.312**	
AP		0.05:0.50			small			0.215	
AP		0.05:0.50			medium			0.544	
AR		0.05:0.50			all			**0.694**	
AR		0.05:0.50			small			0.790	
AR		0.05:0.50			medium			0.589	
**c) Average Precision (AP) and Average Recall (AR) scores for FasterRCNN R50 ALDH1L1 on test 05019 cohort 2**.									
**Metric**	**IoU**			**Area**			**Value**		
AP	0.05:0.50			all			**0.830**		
AP	0.05:0.50			small			0.829		
AP	0.05:0.50			medium			0.890		
AR	0.05:0.50			all			**0.886**		
AR	0.05:0.50			small			0.889		
AR	0.05:0.50			medium			0.893		
**d) Average Precision (AP) and Average Recall (AR) scores for Faster-RCNN R50 GFAP on test 05019 cohort 2**.									
**Metric**		**IoU**			**Area**			**Value**	
AP		0.05:0.50			all			**0.792**	
AP		0.05:0.50			small			0.791	
AP		0.05:0.50			medium			0.926	
AR		0.05:0.50			all			**0.928**	
AR		0.05:0.50			small			0.928	
AR		0.05:0.50			medium			0.988	
**e) Average Precision (AP) and Average Recall (AR) scores for FasterRCNN R50 ALDH1L1 on test 03557**.									
**Metric**	**IoU**			**Area**			**Value**		
AP	0.05:0.50			all			**0.540**		
AP	0.05:0.50			small			0.500		
AP	0.05:0.50			medium			0.865		
AR	0.05:0.50			all			**0.648**		
AR	0.05:0.50			small			0.613		
AR	0.05:0.50			medium			0.924		
**f) Average Precision (AP) and Average Recall (AR) scores for Faster-RCNN R50 GFAP on test 03557**.									
**Metric**		**IoU**			**Area**			**Value**	
AP		0.05:0.50			all			**0.697**	
AP		0.05:0.50			small			0.640	
AP		0.05:0.50			medium			0.890	
AR		0.05:0.50			all			**0.934**	
AR		0.05:0.50			small			0.925	
AR		0.05:0.50			medium			0.958	

The area column refers to the standard COCO pixel area sizes.

## Data Records

We collected a total of 8730 patches, each measuring 500 × 500 pixels, from two distinct histochemical stainings across 16 slides and from 8 different patients. Additional, detailed information could be found in Tables 1, 2. For both stainings, training and test sets derive from different patients, therefore the data splits are patient level stratified. The test sets were annotated by specialists at three levels of expertise: junior, mid-level and expert, with varying years of experience. In total, this dataset contains 103,684 bounding box annotations. All additional information about the annotations is provided in Table 2. The corresponding images and annotations are available in the Figshare^24^ archive associated with this publication.

The dataset comprises three folders: ALDH1L1, GFAP and mmdetection in addition to the individual whole slides. The ALDH1L1 and GFAP folders contain both training and multiple test sets. Annotations are provided as JSON files, detailing consensus and specialist evaluations. All patched image files use the standard PNG format, eliminating the need for specialized viewing tools. The mmdetection folder contains the trained baseline models and their evaluations against the consensus annotations with visualizations and the well defined training pipelines. Raw scans in Aperio (.tif) and MIRAX (.mrxs) formats have been shared alongside the dataset. Each patch includes the name of the original whole-slide image it is coming from. Also, each patch image file’s name contains the top left corner’s x, y location and width and height on the whole slide at the highest resolution. For images with the sub-string *level* in their names, users should extract them at the magnification level specified in their filenames. It’s important to note that users don’t need to undertake these steps; we’ve ensured everything is prepared for immediate use. We provide this information primarily to simplify reproducibility.

## Technical Validation

Quality control was applied multiple times at different stages. Negative control sections were always used during histochemical stainings, in which case no primary antibody was applied on the sections, in order to control for nonspecific binding of the primary antibody. No staining was observed on the negative control sides. Staining quality was checked with a light microscope before scanning. Low quality (e.g. blurred, striped) scans were re-scanned. The scanned images were annotated by mid-level (E.F, T.T, P.H) and expert (I.A.) staff (in case of training) in addition to trainee students (for some test sets) and all consensus annotations were validated and adjusted if needed by an expert neurohistologist (I.A).

## Usage Notes

The patches included in this dataset have been saved as standard PNG format images, while the corresponding annotations have been provided in JSON files using the standard COCO-format^5^. The COCO annotations can be easily accessed and visualized using the Detectron2 library (https://github.com/facebookresearch/detectron2), which also offers flexibility in experimenting with various detection algorithms and includes essential evaluation scripts. The whole slides are compressed for easier handling, for addtional details, refer to Tables 1, 2. The data presented in this dataset is made available under the CC0 4.0 license, providing an open and accessible resource for research and development in the field.

## Data Availability

In order to do the evaluation we made the Python package (https://github.com/qbeer/coco-froc-analysis) accessible. We generated FROC curves with this tool and generally it is possible to use it for binary evaluation for COCO formatted data.
